# De novo *KCNB1* missense variant causing developmental and epileptic encephalopathy: Two case reports

**DOI:** 10.1097/MD.0000000000041236

**Published:** 2025-01-10

**Authors:** Ying Ren, Wandong Hu, Zaifen Gao, Jianguo Shi, Yong Liu, Hongwei Zhang

**Affiliations:** a Epilepsy Center, Children’s Hospital Affiliated to Shandong University, Jinan, China; b Epilepsy Center, Jinan Children’s Hospital, Jinan, China; c Department of Neurology and Endocrinology, Children’s Hospital Affiliated to Shandong University, Jinan, China; d Department of Neurology and Endocrinology, Jinan Children’s Hospital, Jinan, China.

**Keywords:** developmental and epileptic encephalopathy, genotype, *KCNB1* mutation, phenotype

## Abstract

**Rationale:**

Developmental and epileptic encephalopathy (DEE) defines a group of severe and heterogeneous neurodevelopmental disorders. The voltage-gated potassium channel subfamily 2 voltage-gated potassium channel α subunit encoded by the *KCNB1* gene is essential for neuronal excitability. Previous studies have shown that *KCNB1* variants can cause DEE. Herein, we report the cases of 2 children with DEE caused by pathogenic variants in the *KCNB1* gene. Trio whole-exome sequencing identified novel *KCNB1* genotypes, c. 1160C > A and c.1012C > T, which had not been reported previously, in 2 unrelated patients.

**Patient concerns:**

Two children were admitted to our hospital for a detailed evaluation of frequent seizures. And both of these children have abnormal electroencephalogram and brain magnetic resonance imaging results, accompanied by developmental delay.

**Diagnoses:**

A genetic study using trio-whole-exome sequencing confirmed the diagnosis of *KCNB1*-related developmental and epileptic encephalopathy.

**Interventions:**

Both patients accepted the treatment of antiepileptic drugs. 1 patient had seizure remission with a combination of sodium valproate and lamotrigine, and the other was lost to follow-up.

**Outcomes:**

Trio-whole-exome sequencing technology was used to determine the etiology of the 2 children with DEE.

**Lessons:**

This study confirmed that genetic testing provides a basis for the diagnosis of children with abnormal electroencephalogram and brain magnetic resonance imaging findings and developmental delay, and provides data supporting a future phenotype–genotype correlation study.

## 1. Introduction

Developmental and epileptic encephalopathy (DEE) is a severe neurodevelopmental disorder in which abnormal manifestations result from a combination of underlying developmental abnormalities and frequent epileptic activity.^[1]^ Pathogenic variants in ion channel genes are common causes of DEE^[2]^; approximately 25% of monogenic inherited epilepsy cases are associated with ion channel variants.^[3]^ Potassium channels represent the most numerous and complex family of ion channels. These channels are critical to many cellular processes, play an essential role in the pathogenesis of epilepsy, and are effective for targeted therapy.^[3]^ Voltage-gated K^+^ channels regulate the selective flux of K^+^ ions across the cell membrane by opening, closing, or inactivating in response to changes in cell membrane voltage.^[4]^ The voltage-gated potassium channel subfamily 2 (Kv2.1) is primarily responsible for delayed rectifier potassium currents, which are important regulators of the excitability of electrically excited cells including neurones, particularly during high-frequency firing.^[5]^ *KCNB1* encodes the α subunit of Kv2.1. In 2014, de novo pathogenic variants of *KCNB1* have been found to cause infantile-onset epileptic encephalopathy.^[6]^ Despite the rapid development of high-throughput sequencing technology over the past decade, only a few cases of *KCNB1*-related neurodevelopmental disorders have thus far been identified. Nonetheless, the correlation between the *KCNB1* genotype and disease phenotype remains unclear. The general consensus is that pathogenic variants of *KCNB1* may be associated with a broader phenotypic spectrum. Nearly all patients present with early psychomotor retardation and mild-to-severe intellectual disability,^[2,7,8]^ while >80% experience seizures. Sleep disorders, autism, attention deficits, and hyperactivity disorders are also common phenotypes of pathogenic variants of the *KCNB1* gene.^[2,7]^

Herein, we present 2 cases of DEE caused by a heterozygous pathogenic variant of *KCNB1*. As none of the variants detected in this study have been previously reported, this report provides a basis for genotype–phenotype correlation studies of *KCNB1*.

## 2. Case presentation

This study was approved by the institutional review board of the authors, and written informed consent was obtained from all parents.

### Case 1:

**Figure 1.**
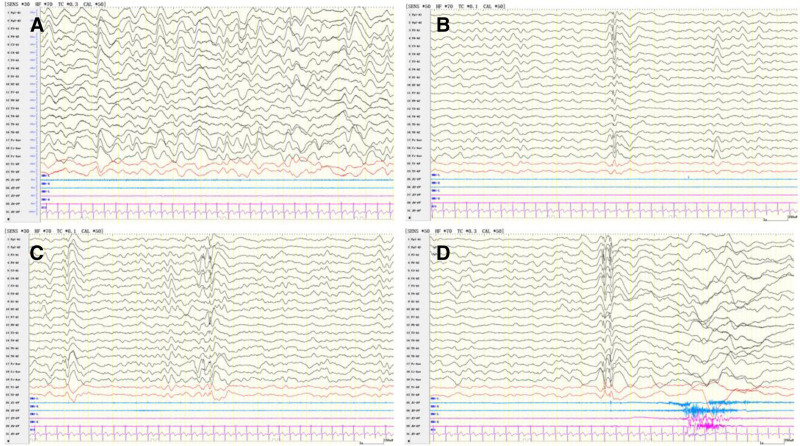
EEG recording for Case 1. (A) Background rhythm; (B,C) interictal epileptic discharges; and (D) myoclonic seizure. EEG = electroencephalography.

**Figure 2.**
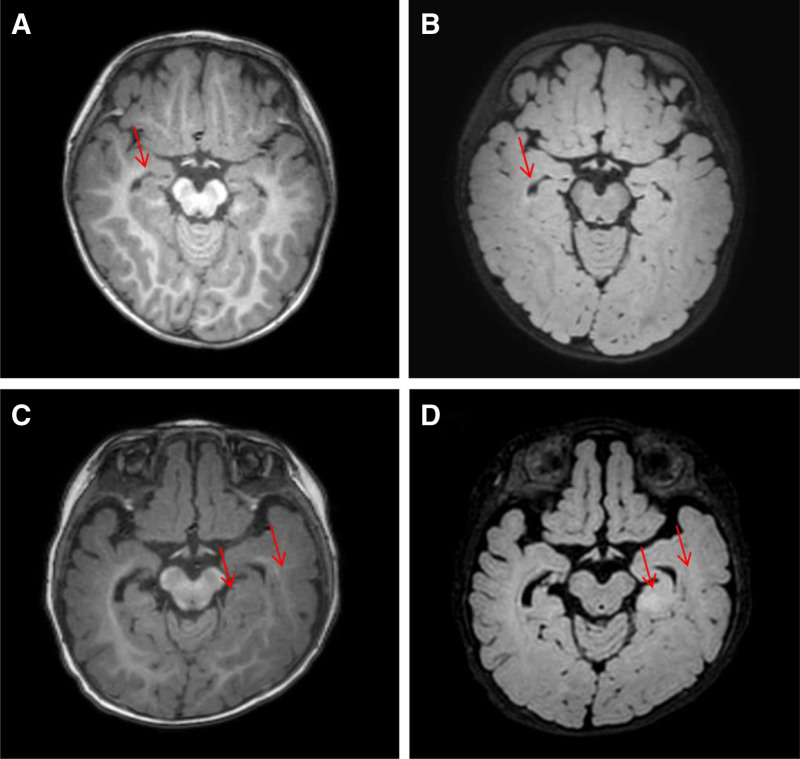
Brain MRI images of the 2 patients. Brain MRI of Patient 1 showing a slightly smaller right hippocampus on T1-weighted imaging (A) and a fluid-attenuated inversion recovery (FLAIR) sequence (B). Brain MRI of Patient 2 showing swelling of the left temporal lobe with abnormal signal intensity suggestive of FCD and an abnormal signal in the left hippocampus on T1-weighted imaging (C) and fluid-attenuated inversion recovery (FLAIR) sequence (D). FCD = focal cortical dysplasia, MRI = magnetic resonance imaging.

**Figure 3.**
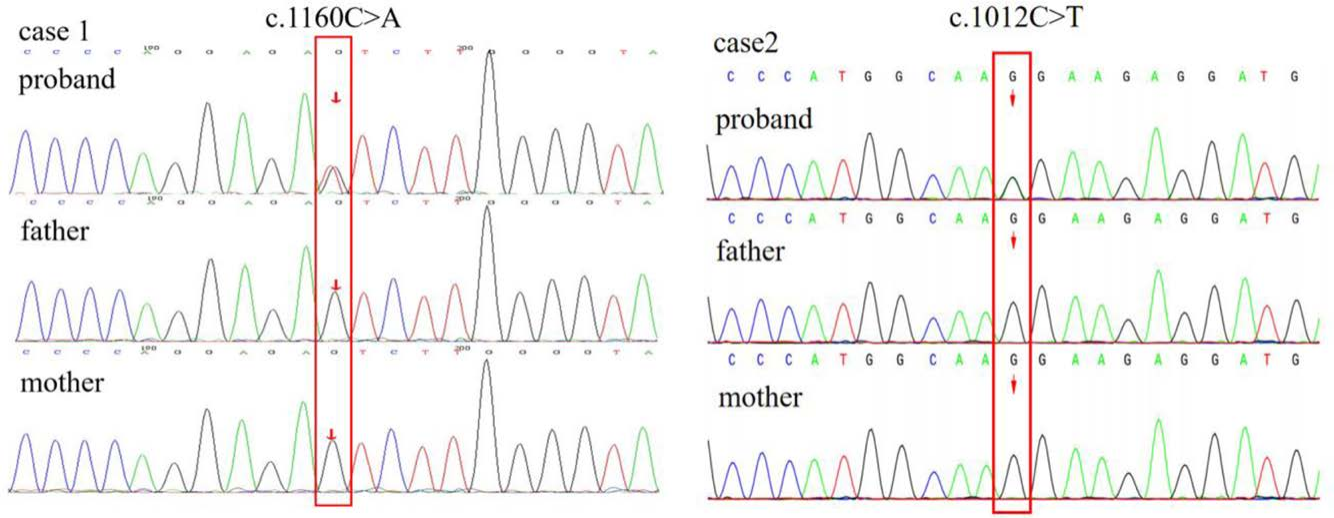
Validation of KCNB1 variants by Sanger sequencing in patients 1 and 2 and their parents.

**Figure 4.**
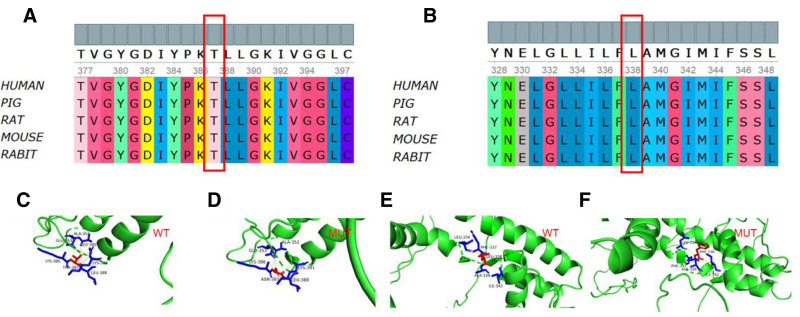
Conservation assessment of *KCNB1* variant sites and KCNB1 protein tertiary structures of the wild-type and mutants. (A) In silico analysis of p.Thr387 in KCNB1 cells showing that p.Thr387 was highly conserved across different species. (B) The in silico analysis of p.Leu338 in KCNB1 revealed that p. Leu338 is highly conserved in different species. (C–F) Three-dimensional structures of wild-type and mutant KCNB1. The p.Thr387 variant altered the structure of the side strands and of the H-bonds. The p.Leu338 mutation altered the side-strand structure.

A 1-year and 4-month-old male child was admitted to our hospital for a detailed evaluation of seizures lasting 3 months. The infant was delivered naturally at term, and had no history of hypoxia or asphyxia, with a birth weight of 3850 g. The parents were healthy and non-consanguineous. The proband was the third child in the family consisting of 2 healthy siblings, and there was no family history of genetic diseases. The patient’s seizure onset occurred at the age of 1 year and 1 month. Seismic seizures were characterized by a nodding head; however, they were initially rare at disease onset and therefore did not attract the parents’ attention. In the third month of disease progression, the seizures clustered and were characterized by upturned eyes, cyanosis of the lips, shaking of the limbs, and nonresponses to calls between 2 and 3 min in duration. Electroencephalography revealed a significantly slow background rhythm, together with generalized epileptiform discharges and myoclonic seizures (Fig. 1). Brain magnetic resonance imaging revealed gliosis in the posterior right lateral ventricle, and a slightly smaller right hippocampus (Fig. 2A and B). The Gesell developmental schedule revealed moderate developmental delay in language and mild developmental delay in other areas. Routine blood tests, liver and kidney function tests, and inherited metabolic disease tests were all normal. The patient was subsequently diagnosed with epilepsy; however, the etiology of his seizures was unclear. Therefore, DNA was subsequently extracted from the peripheral blood of the proband and his parents, and was analyzed using trio-whole-exome sequencing. Taking into account the phenotypes and characteristics of the variants, we prioritized a heterozygous variant de novo variant in exon 2 (c.1160C > A, p.Thr387Asn), which has not been previously reported. According to the American College of Medical Genetics and Genomics guidelines, this variant was considered likely pathogenic.^[9]^ The mutation was confirmed in the DNA of the proband using Sanger sequencing, and was excluded from the parental samples (Fig. 3). Conservation analysis revealed that the threonine residue at position 387 (p.Thr387) was highly conserved in multiple species (Fig. 4A). Structural modeling revealed changes in the side-strand structure and in the H-bond at residue 387, in which threonine was substituted with asparagine (Fig. 4C and D). The patient was treated with sodium valproate and lamotrigine. The most recent follow-up was in March 2024, during which he was reported to be seizure-free for 12 months. The child presented a language developmental delay, although no formal psychological assessment had been performed at the time of evaluation. He was able to understand simple commands and could speak words such as “baba” and “mama.”

### Case 2:

A 7-month-old female infant was admitted because of seizures persisting for 2 months. The proband was born to healthy non-consanguineous parents. No family history or exposure to teratogens was reported. She was born naturally at term, and had no history of hypoxia or asphyxia. Birthweight was 2900 g, with no evidence of congenital or dysplastic features at birth. She was the first child of the family. The onset of seizures occurred at the age of 5 months, characterized by upturned eyes and generalized hypotonia and a duration of approximately 30 min. The frequency of the seizures was once per month. The patient underwent a 24-h video-electroencephalogram examination, and the results revealed many sharp waves and sharp slow-wave synchronous/asynchronous discharge in both occipital areas. Brain magnetic resonance imaging revealed swelling of the left temporal lobe with abnormal signal intensity (focal cortical dysplasia, FCD), with abnormal signals observed in the left hippocampus (Fig. 2C and D). The trio-whole-exome sequencing revealed a heterozygous de novo variant of *KCNB1* (c.1012C > T, p.Leu338Phe). According to American College of Medical Genetics and Genomics guidelines, this variant was considered likely pathogenic.^[9]^ However, this variant has not been described previously. The de novo mutation was confirmed using Sanger sequencing (Fig. 3). Conservation analysis revealed that the leucine at site 338 (p.Leu338) was highly conserved across multiple species (Fig. 4B). Structural modeling revealed changes in the side-strand structure at residue 338, in which leucine was substituted with phenylalanine (Fig. 4E and F). The patient presented with a global developmental delay, although she did not complete the developmental tests. Her developmental milestones occurred significantly later than those of the corresponding age norms. The patient was treated with levetiracetam monotherapy, and was seizure-free at her most recent visit. As the parents discontinued treatment at our hospital, no follow-up was performed.

## 3. Discussion

DEE associated with variants of the *KCNB1* gene presents an autosomal dominant inheritance. To date, 98 missense variants in *KCNB1* have been reported. Herein, we describe the cases of 2 children with DEE associated with heterozygous de novo *KCNB1* missense variants; neither of which has been reported previously. Patient 1 achieved seizure remission with a combination of valproate and lamotrigine treatment, which represents a reliable option for the treatment of *KCNB1*-related epilepsy. However, due to our limited sample size, a large number of clinical trials are still required to verify the treatment strategy for these patients.

*KCNB1*(OMIM# 600397) is a potassium channel gene with 2 exons located on chromosome 20q13.3. *KCNB1* encodes the α subunit of the Kv2.1 transported containing 858 amino acid residues. This subunit has 6 transmembrane domains (S1–S6), of which S1–S4 constitutes a voltage-sensing domain, while S5–S6 constitutes a pore domain that can selectively filter potassium ions. In vivo, tetramers that assemble only by α subunits are called homotetrameric Kv2.1 channels, whereas tetramers assembled by both α subunits and other families of channel-forming subunits are referred to as heterotetrameric Kv2.1 channels.^[8]^ Homotetrameric Kv2.1 channels contribute to a significant fraction of delayed rectifier potassium currents in various neuronal subtypes, including pyramidal neurones in the hippocampus and cortex, are more complex, and depend on the subunit composition of the channel.^[8,10]^ In vitro and in vivo experiments have verified that the pathogenic mechanism of *KCNB1*-related diseases involves channel hypofunction, altered voltage dependence, reduced protein expression, or cell surface trafficking due to *KCNB1* gene variants.^[5,11]^ Kv2.1 also forms complexes with integrins that are essential for the migration of glutamatergic neurones during prenatal brain development and regulates migration through nonionic functions.^[12]^

The 2 cases presented herein show some similarities to other cases described in the literature, including developmental delay, abnormal electroencephalograms, and seizures. However, certain unique points must be addressed. Minor dysmorphic features were observed in 14 of 36 patients with *KCNB1* variants reported by Bar et al.^[2]^ Deformity features were most obvious in the oldest patient (age 34 years).^[2]^ Due to the young age of our patients, no obvious behavioral abnormalities or dysmorphic characteristics were observed. The evolution of morphology over time requires attention. Both patients evaluated at our center had abnormal brain imaging findings: hippocampal damage and FCD, which is not a common phenotype. Case 2 had an FCD, as did another patient harboring a *KCNB1* variant in a previously reported case.^[2]^ Pathogenic mutations in genes encoding ion channels have been shown to result in FCD.^[11]^ Therefore, while FCD is rare in patients with *KCNB1* variants, it is reasonable to assume that pathogenic variants in *KCNB1* are associated with FCD. Subtle volume losses in the hippocampus have been observed in several cases.^[8,13,14]^ Both cases in this report presented with hippocampal damage, suggesting that this phenotype does not occur by accident, but is caused by variants in the *KCNB1* gene. However, the phenotypes of *KCNB1*-related diseases are highly heterogeneous. Currently, genotype–phenotype correlations cannot be established based on the localization of missense variants in protein domains. Further experimental studies are required to understand the pathophysiology underlying this phenotypic heterogeneity.

Approximately 70% of *KCNB1*-related epilepsy cases are drug refractory.^[2]^ Patient 1 in the present report was initially treated with sodium valproate monotherapy, and generalized myoclonic seizures were reduced by >50%. Upturned eyes, cyanosis of the lips, shaking of the limbs, and nonresponse to calls were not observed. Valproate has the broadest spectrum of anticonvulsant effects among all the currently-available antiepileptic drugs. Valproic acid can enhance the synthesis and release of γ-aminobutyric acid, reduce the release of excitatory molecules, and obtain attenuation of high-frequency neuronal firing by blocking voltage-gated ion channels, including sodium, potassium, and calcium channels.^[15]^ Our patient did not achieve complete remission of seizures with oral sodium valproate monotherapy; therefore, lamotrigine was administered as a co-antiseizure. After gradual adjustment of the drug dose, the patient remained seizure-free for 1 year. Previous studies have suggested that a combination of lamotrigine and sodium valproate should be considered in patients with refractory epilepsy, as its efficacy appears to be independent of seizure type or demographics of the patient.^[16]^ In summary, our treatment approach may be considered as a reliable option for the treatment of *KCNB1*-related epilepsy.

## 4. Conclusion

We retrospectively analyzed the phenotypes and genotypes of 2 unrelated patients with variants of *KCNB1.* Neither of the 2 *KCNB1* variant sites has been previously described. This study provides data supporting a future phenotype–genotype correlation study, and further underscores that genetic testing should be performed as early as possible in children with DEE to provide accurate genetic counseling and inform parents of the disease course.

## Acknowledgments

The authors thank the parents of the children involved in this study and contributors to the study who are not included in the author list. We gratefully acknowledge support from the Shandong Children’s Health and Disease Clinical Research Center.

## Author contributions

**Data curation:** Ying Ren.

**Investigation:** Wandong Hu.

**Writing – original draft:** Ying Ren.

**Writing – review & editing:** Zaifen Gao, Jianguo Shi, Yong Liu, Hongwei Zhang.
